# Development and validation of a nomogram to predict suicidal behavior in female patients with mood disorder

**DOI:** 10.3389/fpsyt.2023.1212579

**Published:** 2023-07-07

**Authors:** Sixiang Liang, Xinyu Liu, Dan Li, Jinhe Zhang, Guangwei Zhao, Hongye Yu, Xixi Zhao, Sha Sha

**Affiliations:** ^1^National Clinical Research Center for Mental Disorders and National Center for Mental Disorders, Beijing Key Laboratory of Mental Disorders, Beijing Anding Hospital, Capital Medical University, Beijing, China; ^2^Advanced Innovation Center for Human Brain Protection, Capital Medical University, Beijing, China

**Keywords:** suicidal behavior, mood disorder, predictive model, nomogram, validation

## Abstract

**Introduction:**

This study aims to explore the risk factors associated with suicidal behavior and establish predictive models in female patients with mood disorders, specifically using a nomogram of the least absolute shrinkage and selection operator (LASSO) regression.

**Methods:**

A cross-sectional survey was conducted among 396 female individuals diagnosed with mood disorders (F30-F39) according to the International Classification of Diseases and Related Health Problems 10th Revision (ICD-10). The study utilized the Chi-Squared Test, *t*-test, and the Wilcoxon Rank-Sum Test to assess differences in demographic information and clinical characteristics between the two groups. Logistic LASSO Regression Analyses were utilized to identify the risk factors associated with suicidal behavior. A nomogram was constructed to develop a prediction model. The accuracy of the prediction model was evaluated using a Receiver Operating Characteristic (ROC) curve.

**Result:**

The LASSO regression analysis showed that psychotic symptoms at first-episode (*β* = 0.27), social dysfunction (*β* = 1.82), and somatic disease (*β* = 1.03) increased the risk of suicidal behavior. Conversely, BMI (*β* = −0.03), age of onset (*β* = −0.02), polarity at onset (*β* = −1.21), and number of hospitalizations (*β* = −0.18) decreased the risk of suicidal behavior. The area under ROC curve (AUC) of the nomogram predicting SB was 0.778 (95%CI: 0.730–0.827, *p* < 0.001).

**Conclusion:**

The nomogram based on demographic and clinical characteristics can predict suicidal behavior risk in Chinese female patients with mood disorders.

## 1. Introduction

Suicide is a significant global public health issue, causing 703,000 deaths annually worldwide (1). It has become one of the leading causes of death worldwide, placing a severe burden on public health, society, and families (2). The suicide rate among Chinese individuals is 6.7%, with 8.6 and 4.8% for males and females, respectively (3). Suicidal behavior (SB) includes suicidal ideations, attempts, and completed suicidal acts (4). SB outnumbered suicide deaths by 20–30 times (5, 6), which further increases the social and economic burden (7).

The global age-standardized suicide rate is 9.0 per 100,000 people (3). Patients with mental disorders are more likely to engage in SB (8). Depression is the leading cause of suicide deaths worldwide, with depressive disorder and other mood disorders reported in half of all suicides, with a reported 20-fold increased risk compared to the general population (1, 9).

Previous studies have found numerous factors associated with SB, such as experiencing adverse life events (10, 11), unemployment or lower economic status, female, younger age, and suicide of a close relative (12–14). Health risk behaviors have also been found to be associated with suicidal behaviors, including physical inactivity and sedentary behavior (15–17), substance use (alcohol, tobacco, drugs) (18–20), and passive smoking (21, 22). However, no studies have developed predictive models of SB based on demographic information and clinical characteristics of patients with mood disorders. Therefore, it is critical to identify the risk of suicide reasonably and prevent it to ensure that the tragedy of suicide does not continue to cost lives.

Although more males die by suicide than females (3), females were 2–3 times more likely to have SB than males (12, 13, 23). The higher rate of SB may be explained by gender role socialization theory, which states that females are perceived as dependent and indecisive and express their stress through rumination, leading to higher rates of suicidal behaviors (12). Therefore, this study was conducted only in female patients to obtain a more rigorous prediction model.

The aim of this study was to explore risk factors associated with suicidal behavior to establish predictive models in female patients with mood disorders, specifically using the nomogram of the least absolute shrinkage and selection operator (LASSO) regression.

## 2. Materials and methods

### 2.1. Participants

In this study, we conducted a cross-sectional retrospective analysis using data extracted from the inpatient medical records of Beijing An Ding Hospital between 2019 and 2021. All participants were independently diagnosed by at least two attending psychiatrists and met the mood disorder diagnostic groups (F30-F39) based on the International Classification of Diseases and Related Health Problems 10th Revision (ICD-10) (24). To protect the privacy of participants, personal information was erased. All patients were informed and agreed in advance that their medical record information could be shared anonymously for the purpose of the study. The study protocol received approval from the Ethics Committee of Beijing An Ding Hospital in 2018. Written informed consent was obtained from all participants for this study.

396 female hospitalized patients with MD were included in this study. We reviewed patients’ complete medical histories and excluded those with other psychiatric disorders, including schizophrenia, schizoaffective disorder, personality disorders, intellectual disabilities, and alcohol or drug abuse. The use of medications such as antidepressants and atypical antipsychotics did not affect participants’ inclusion in the study.

### 2.2. Materials

We collected sociodemographic information and clinical characteristics, including age, years of education, illness duration, age of onset, duration of first onset, medication duration, number of hospitalizations, body mass index (BMI), marital status, occupation, family history, social dysfunction, income, place of residence, polarity at onset, presence of psychotic symptoms at first episode, and comorbid somatic diseases.

### 2.3. Suicidal behavior assessment

For questions related to suicidal behavior, we utilized the following lifetime variables: (1) suicidal ideation, (2) suicidal planning, and (3) suicide attempts. Individuals who responded positively to any of these three variables were classified as the suicidal behavior (SB) group, while those who did not were categorized as the non-SB (SB-N) group. The assessments of SB were completed by psychiatrists and were documented in the medical records.

### 2.4. Social dysfunction assessment

Social functioning refers to an individual’s ability to fulfill various social roles in society and their actual social performance (25). We evaluated patients’ social functioning using the Personal and Social Performance (PSP) Scale (26), which assesses socially useful activities (e.g., work and study), personal and social relationships, self-care, and disruptive and aggressive behavior (26). Patients were categorized as having social dysfunction or not based on a cutoff score of 70 points.

### 2.5. Statistical analyses

Continuous variables were described using mean and standard deviation, while categorical variables were described using counts and percentages. Differences in demographic and clinical characteristics between SB and SB-N were tested using Chi-square and Wilcoxon rank-sum tests. Logistic LASSO regression analysis was then used to identify risk factors for SB based on demographic and clinical characteristics. The LASSO model is a shrinkage method that automatically selects variables to eliminate non-influential variables, produce a more relevant and interpretable set of predictors, and avoid over-fitting. We used ten-fold cross-validation to choose the penalty term λ and computed the binomial deviation of the test data as a measure of the predictive performance of the fitted model. The standard errors of the LASSO coefficients were obtained *via* bootstrapping within the primary sampling unit and strata. In the LASSO regression, we included 18 variables. We selected the left λ value as it had the smallest binomial deviance.

Using the risk factors screened by LASSO regression, a predictive nomogram was created, assigning each predictor an initial score ranging from 0 to 100. The scores for each risk factor were then summed to obtain a total score, which was finally converted to the probability of having suicidal behavior (from 0 to 100%). In the prediction model, we defined patients with depression as the primary form of onset as 1, mania as the primary form of onset as 2, and delusion as the primary form of onset as 3 for the polarity at onset variable. For patients without social dysfunction, psychotic symptoms at first-episode, and somatic disease, we defined them as 0, and 1 otherwise. The performance of the nomogram was evaluated by Harrell’s concordance index (C-index) and the calibration plot. A C-index >0.7 reflects the good-fit characteristics of the prediction model in general. A receiver operating characteristic (ROC) curve was also calculated to evaluate the predictive ability of the model.

Statistical analyses were conducted using STATA software Special Release 14.0 (Stata Corp, TX). LASSO regression, nomogram, and ROC curve were generated using R-language (version 3.5.2). The LASSO logistic regression model was generated with the “glmnet” package. The nomogram and calibration curve were generated with the “rms” package, and the ROC curves were plotted with the “pROC” package. A significance level of *p* < 0.05 was used.

## 3. Results

### 3.1. Demographic characteristics

The present analysis included 396 female patients who were hospitalized (mean age: 35.81 years old, SD =15.65). Of these patients, 32.6% had SB. The SB group and the SB-N group differed significantly in terms of age, duration, age of onset, number of hospitalizations, BMI, social dysfunction, and polarity at onset (Table 1).

**Table 1:** Demographic information and clinical characteristics.

	SB (*n* = 129)	SB-N (*n* = 267)	*p*	χ^2^/z
Age (years)^*^	32.02 (16.17)	37.64 (15.09)	<0.001	3.75
Educational years (years)	13.10 (2.73)	13.47 (2.79)	0.117	1.57
Duration (months)	81.84 (106.37)	108.07 (108.86)	<0.001	3.33
Age of onset (years)	24.40 (13.40)	28.42 (13.43)	<0.001	3.79
Duration of first onset (months)	3.59 (3.95)	3.92 (5.32)	0.953	−0.06
medication time (months)	6.24 (11.36)	6.98 (12.34)	0.611	0.51
Number of hospitalizations	1.66 (1.80)	2.14 (2.06)	0.001	3.24
BMI (kg/m^2^)	23.43 (5.58)	24.68 (5.83)	0.038	2.07
Marital status (*n*, %)			0.092	4.78
unmarried	84 (65.1%)	145 (54.3%)		
Divorce/widowhood	7 (5.4%)	26 (9.7%)		
married	38 (29.5%)	96 (36%)		
Job (*n*, %)			0.126	4.14
Manual labour	5 (3.9%)	13 (4.9%)		
Mental labour	55 (42.6%)	86 (32.2%)		
Unemployed	69 (53.5%)	168 (62.9%)		
Family history (*n*, %)	35 (27.1%)	81 (30.3%)	0.511	0.43
Social dysfunction	103 (79.84%)	117 (43.82%)	<0.001	45.72
Income (*n*, %)			0.606	1.84
<¥1,000	23 (17.8%)	36 (13.5%)		
¥1,000–2,999	31 (24%)	77 (28.8%)		
¥3,000–5,000	34 (26.4%)	70 (26.2%)		
>¥5,000	41 (31.8%)	84 (31.5%)		
Residence (*n*, %)			0.343	2.14
City	101 (78.3%)	196 (73.4%)		
Villages and towns	4 (3.1%)	17 (6.4%)		
Countryside	24 (18.6%)	54 (20.2%)		
Polarity at onset (*n*, %)			<0.001	16.11
Depression	119 (92.2%)	201 (75.3%)		
Mania	7 (5.4%)	50 (18.7%)		
Delusion	3 (2.3%)	16 (6%)		
Psychotic symptom at first-episode (*n*, %)	47 (36.4%)	78 (29.2%)	0.147	2.10
Somatic disease (*n*, %)	126 (97.7%)	254 (95.1%)	0.228	1.45

^*^Mean (SD).

### 3.2. Predictors selection and development of an individualized prediction model

**Figure 1:**
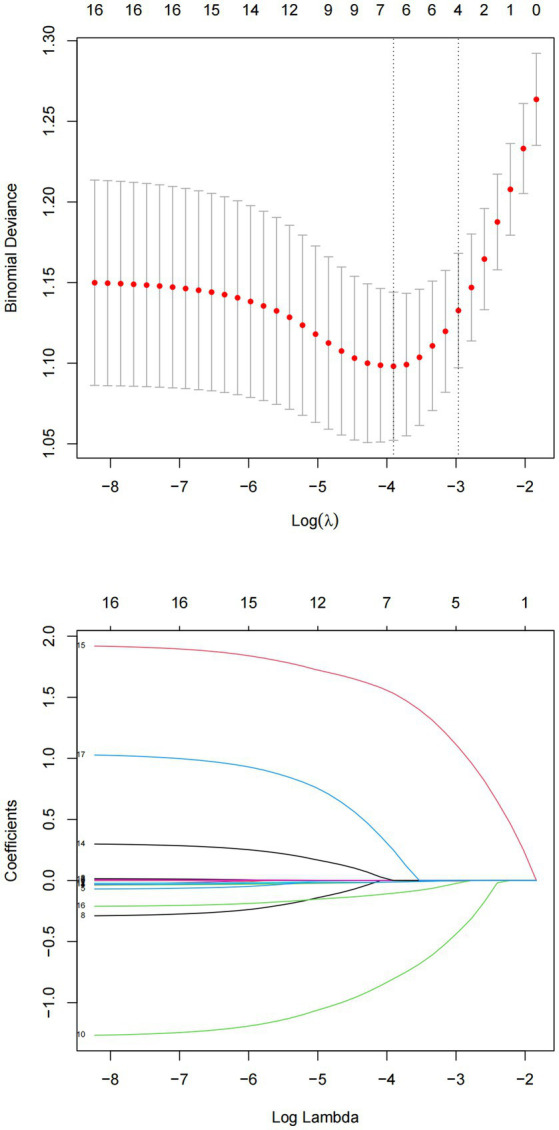
Cross validation plot for the penalty term and plots for LASSO regression over different values of the penalty parameter.

After the LASSO regression selection, 7 variables remained as significant predictors of SB (Figure 1). These variables were BMI, age of onset, polarity at onset, psychotic symptom at first-episode, social dysfunction, number of hospitalizations, and somatic disease. The estimated coefficients for LASSO regression are presented in Table 2. Psychotic symptom at first-episode (*β* = 0.27), social dysfunction (*β* = 1.82), and somatic disease (*β* = 1.03) increased the risk of SB, while BMI (*β* = −0.03), age of onset (*β* = −0.02), polarity at onset (*β* = −1.21), and number of hospitalizations (*β* = −0.18) decreased the risk of SB.

**Table 2:** The estimated coefficients for LASSO regression.

Variables	Coefficients	Std. error
BMI	−0.03	0.02
Social dysfunction	1.82	0.27
Polarity at onset	−1.21	0.32
Psychotic symptom at first-episode	0.27	0.27
Age of onset	−0.02	0.01
Number of hospitalizations	−0.18	0.07
Somatic disease	1.03	0.71

### 3.3. Prediction model

**Figure 2:**
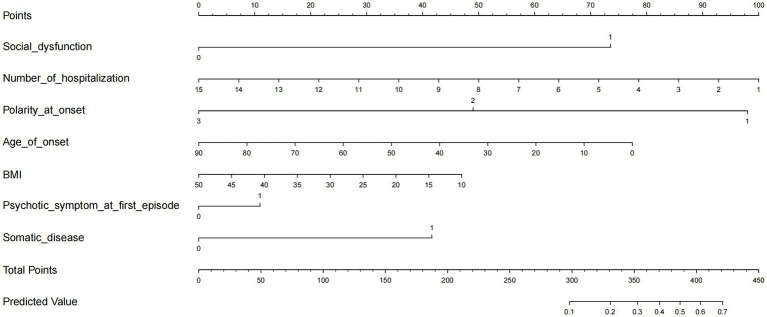
The prediction nomogram of risk factors for SB in female patients with mood disorder.

We developed a nomogram model to predict the risk of SB based on the significant factors identified in the LASSO regression analyses, including BMI, age of onset, polarity at onset, psychotic symptom at first-episode, social dysfunction, number of hospitalizations, and somatic disease (Figure 2).

**Figure 3:**
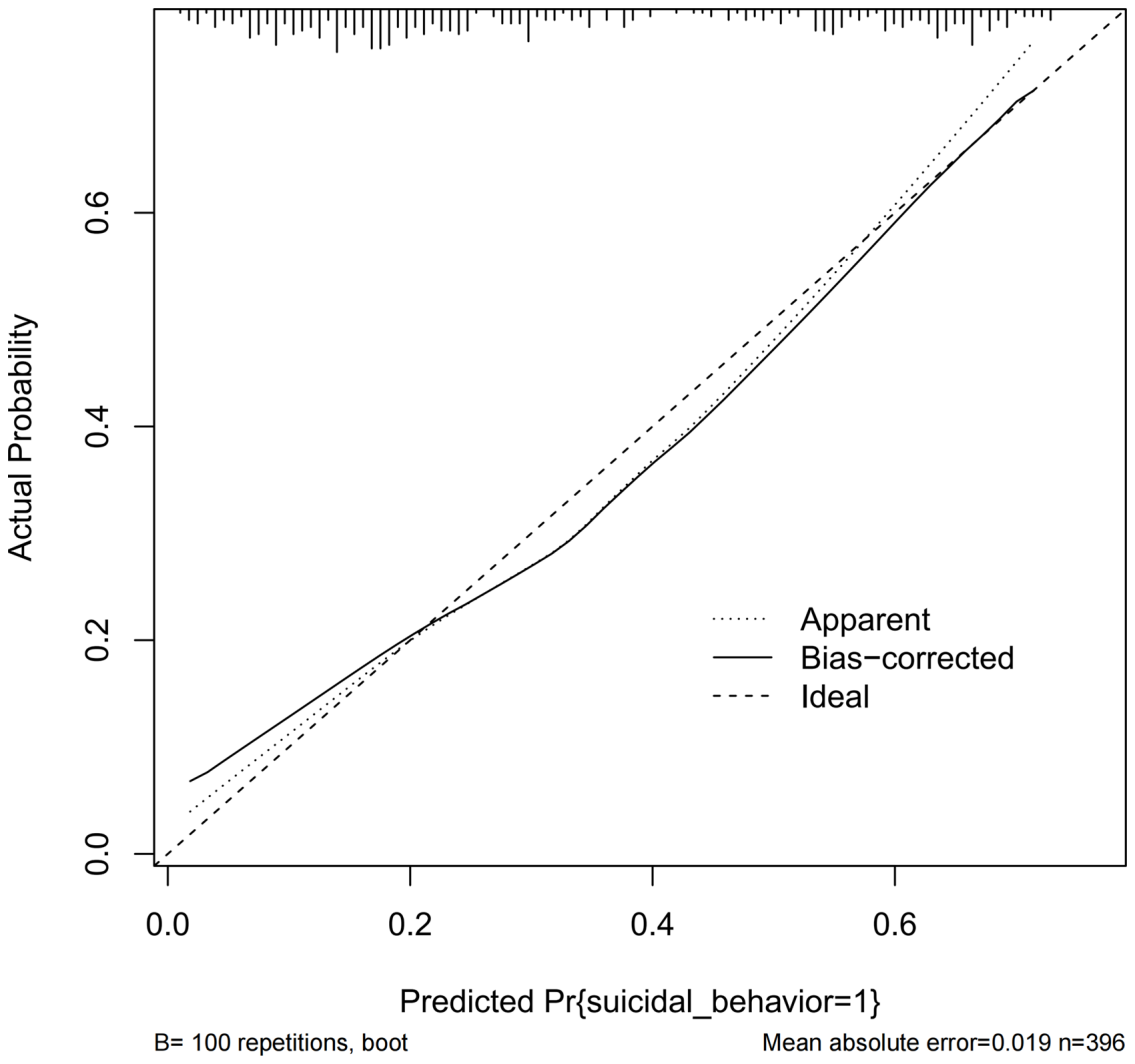
The logistic calibration curve of the prediction nomograms of risk factors for SB in female patients with mood disorder.

**Figure 4:**
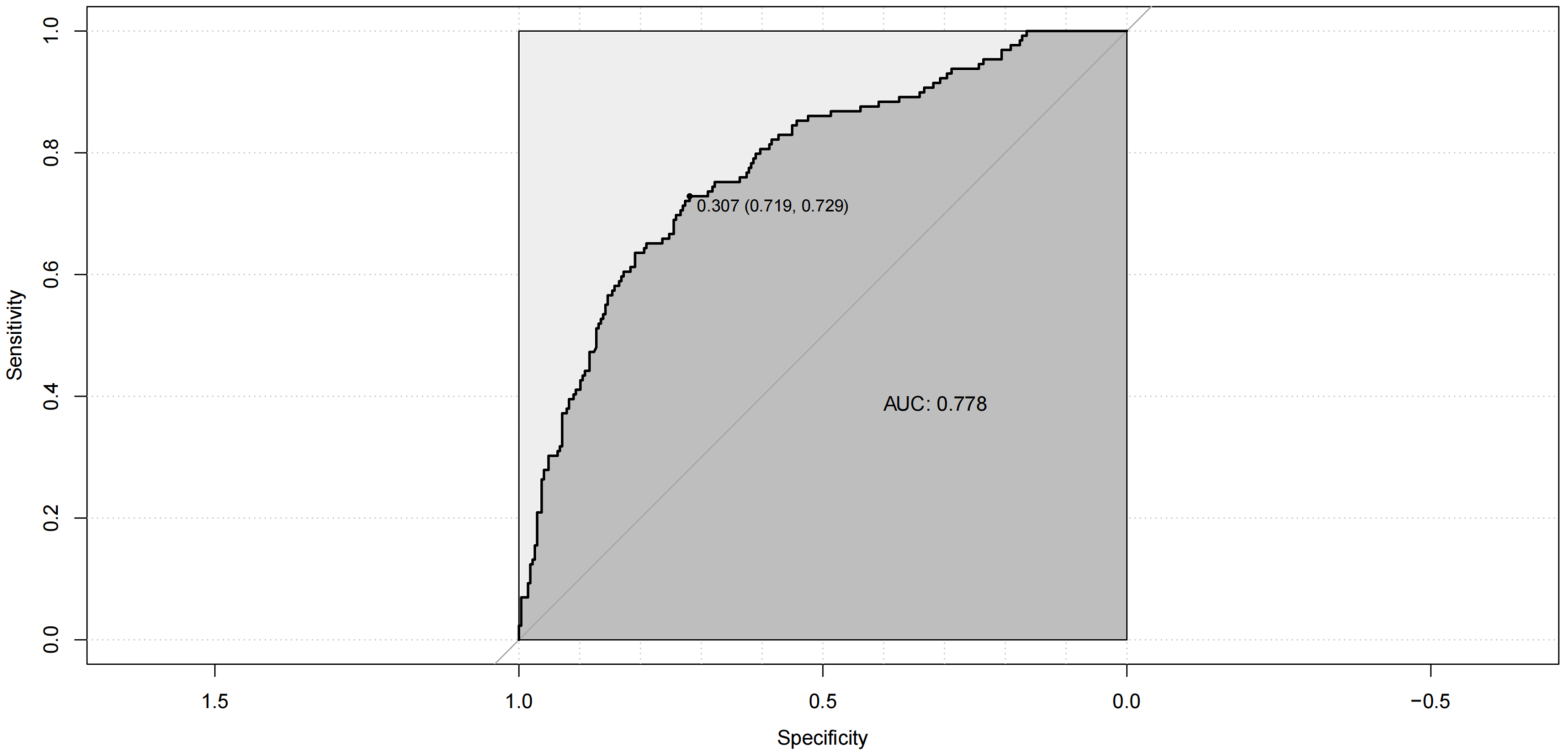
ROC curve for the prediction nomogram of risk factors for SB.

The calibration curve of the nomogram demonstrated good agreement between predicted and observed risk of suicidal behavior. The C-index of the nomogram was 0.778, and it was 0.761 in the internal bootstrap validation sets (Figure 3). The prediction accuracy was further confirmed by the ROC curve, with an AUC of 0.778 (95% CI: 0.730–0.827, *p* < 0.001) (Figure 4).

## 4. Discussion

In this study, we investigated the association between demographic information, clinical characteristics, and the risk of SB in female patients with MD. Using LASSO regression, we eliminated irrelevant variables and found that social dysfunction, number of hospitalizations, polarity at onset, age at onset, BMI, psychotic symptoms at first onset, and somatic disease were significantly associated with SB. Furthermore, we developed a nomogram to quantify the risk of SB. To the best of our knowledge, this is the first study to use LASSO regression and a nomogram to predict the risk of SB in patients with MD.

Social functioning, a crucial skill for human survival and well-being, has been associated with serious health outcomes and premature death in patients with mood disorders (27, 28). Our study found an association between social dysfunction and suicidal behavior (SB) in patients with mood disorders, which is consistent with previous research (29, 30). Furthermore, our results indicate a higher risk of suicide in patients with social dysfunction. Social dysfunction is reflected in various aspects of socialization, including difficulties in dealing with social stress, resolving social problems, and recognizing emotions in others. Deficits in social cognition may lead to communication deficits, disrupt interpersonal relationships, and impair social support. Suicidal individuals have difficulty establishing and maintaining relationships, indicating poor social competence (31). Impaired social functioning is also reflected in reduced social support, which acts as a cushion and protects people from stressors. People who attempted suicide report lower levels of belonging, self-esteem, tangible support, and problem-solving skills compared to those with non-suicidal depression and non-psychotic disorders. Attempters are less likely to have close friends, and they do not participate in any volunteer activities. Narrow social networks, sparse social activities, and perceived isolation have been associated with mental health problems (32, 33). Therefore, social dysfunction may be particularly likely to lead to SB in patients with few meaningful relationships.

Hospitalization is an important tool in the care of mental illness, providing thorough assessment, intensive care, and a range of treatments that are difficult to administer in an outpatient setting (34). Our study found that the risk of suicidal behavior decreased with increasing numbers of hospitalizations, suggesting that hospitalization can significantly improve patients’ emotional symptoms and indirectly reduce suicidal behavior. Previous studies have reached disparate conclusions about the relationship between hospitalization and suicide risk. Some studies have shown that patients with shorter hospital treatment have a higher risk of suicide (35), indicating that hospitalization treatment has a role in suicide prevention. Two early randomized controlled trials examining the influence of hospitalization on suicide were inconclusive (36, 37), but these studies are not representative of the current situation, as psychiatric treatment and rehabilitation techniques have improved significantly since then. Previous studies have shown that hospital treatment can be stigmatizing, traumatic, and coercive, leading to a loss of social support and social roles, and even violence (38–43). However, with the development of psychiatric hospitals, the negative labels of being stigmatized and humiliated have reduced, and patients’ sense of stigma about inpatient treatment has improved significantly. The effect of hospitalization on patients’ risk of suicide may be related to the level of psychiatric treatment in different regions, and further studies with large samples are needed in the future. Therefore, clinicians should help patients maintain social roles and social support during hospitalization, reduce patients’ stigma, and develop specialized suicide prevention strategies to maximize the benefits of hospitalization.

The present study has confirmed that rates of suicidal behavior increase in patients with onset depression. This finding is consistent with previous research (44–46) that has shown a higher lifetime suicide attempt rate in patients with depressive polarity at onset than in those with manic polarity at onset. This suggests that more intensive prevention and management of depressive episodes is needed in patients with mood disorders to reduce the associated burden and impairment. Earlier onset bipolar disorder has been associated with more frequent suicidal ideation (47) and a greater likelihood of attempting suicide over a lifetime (48–50). A meta-analysis has shown that individuals with an earlier age of onset are significantly more likely to attempt suicide than those with a later age of onset (51). In contrast, late-onset depression patients have less severe depressive symptoms and suicidal ideation (52) than early-onset depression patients. Additionally, most of the late-onset patients who died by suicide were male (53). Therefore, among female patients with mood disorders, early age of onset may be a risk factor for suicidal behavior and requires early identification and intervention. However, the higher risk of suicidal behavior in patients with an earlier age of onset may also be the result of a longer duration of untreated depression (51). Further studies are needed to exclude the effect of duration.

The study found that higher BMI could be a possible protective factor for suicidal behavior, possibly due to the significant weight gain produced by antipsychotic drugs. These drugs may prevent suicide deaths by improving mental stability, and weight gain may be a side effect (54). Being overweight is typically associated with an increased risk of chronic diseases, such as hypertension, cardiovascular disease, type 2 diabetes, and osteoarthritis (55, 56). Additionally, overweight or obesity is associated with social stigma and negative attitudes in some cultures (57–59), and often accompanied by poor mental health and quality of life (60, 61). Therefore, it is often assumed that those with a higher BMI may be at a higher risk for suicidal behavior. However, this is not the case. Prospective cohort studies have found that most people who are overweight or obese have a lower risk of suicide or attempted suicide (62–64), and the risk of suicide attempts decreases as BMI increases (65, 66). A study that followed 4,930 women for 16 years found that those with a BMI <20 kg/m2 had a significantly greater risk of suicide attempts and suicide than women with a BMI of 20–24.9 kg/m2. Half of the women in the cohort had a BMI >25 kg/m2 and a somewhat lower risk than those with a BMI of 20–24.9 kg/m2, but the reduction in risk was not strongly related to the level of BMI (67). Therefore, unlike most diseases, being obese may help alleviate suicidal behavior, and the exact reasons for this need to be further explored in the future.

Although psychotic symptoms are not common in mood disorders (68), studies have found that suicidal ideation and suicide attempts are more common in patients with psychotic depression than in those without, and this cannot be attributed to more severe depressive symptoms (69–71). Among patients with bipolar disorder, having at least one lifetime psychotic episode is associated with overall more severe illness and a higher lifetime rate of suicide attempts (72). In our study, the results of the LASSO regression showed that patients with psychotic symptoms at first episode should receive more attention to prevent suicidal behavior.

Our results indicate that somatic diseases may exacerbate the risk of suicidal behavior. Previous studies have shown that certain physical illnesses, such as AIDS and lung disease (73), and cancer (74), not only have a higher rate of comorbid psychiatric disorders, but the physical condition itself may also be a risk factor for suicidal behavior independent of the comorbid psychiatric disorder (73–76). However, some disorders, including diabetes, hypertension, and arthritis, have inconsistent findings regarding their risk for suicidal behavior (73, 74, 76). A German study of 4,181 patients showed that among people with mood disorders, respiratory disease and hypertension were associated with suicidal behavior, independent of the effects of comorbid psychiatric disorders (77). Clinicians should recognize the risk of somatic diseases contributing to suicidal behavior. However, our study did not explore the specific somatic diseases associated with suicidal behavior, and further research is needed to identify which diseases are predictors of suicidal behavior.

To our knowledge, this is the first large sample study to accurately predict suicidal behavior in patients with mood disorders using LASSO regression analysis based on their medical history. We also verified the stability and accuracy of the model using different methods. However, there were some limitations to this study. Firstly, the retrospective design of the study may be biased by recall, and more rigorous study protocols are needed to explore the risk factors for suicidal behavior. Secondly, since this study was cross-sectional, no causal relationship could be established, and more well-designed longitudinal studies are needed in the future. Thirdly, we only conducted the predictive model in female patients because females are more likely to exhibit suicidal behavior (12, 13, 23). However, patients of all genders will be further explored in the future. Finally, The AUC of the ROC curve in our study was 0.778, and the prediction accuracy of the model was not very high, so it should be used with caution in the clinical practice. Subsequent studies can choose methods such as machine learning to create prediction models based on our study to improve the prediction accuracy of suicidal behavior in mood disorder patients.

In conclusion, we found that social dysfunction, number of hospitalizations, polarity at onset, age at onset, BMI, psychotic symptoms at first onset, and somatic disease may be risk factors for suicidal behavior in female patients with mood disorders. Based on these findings, we established a practical nomogram that may predict suicidal behavior and help psychiatrists make individualized treatment plans, improve follow-up management strategies, and prevent suicide more effectively.

## Data availability statement

The raw data supporting the conclusions of this article will be made available by the authors, without undue reservation.

## Ethics statement

The studies involving human participants were reviewed and approved by the Ethics Committee of Beijing An Ding Hospital. The patients/participants provided their written informed consent to participate in this study.

## Author contributions

XL and JZ carried out the recruitment of patients and performed the statistical analysis. SL and DL wrote the manuscript. HY and GZ performed the data processing and analysis, and plotted. SS supervised the entire study. SL, XL, DL, JZ, GZ, HY, XZ, and SS had full access to all study data and analyses, participated in preparing this report. All authors contributed to the article and approved the submitted version.

## Funding

This study was funded by the Beijing Municipal Administration of Hospitals Incubating Program (PX2021068) and Beijing Anding Hospital, Capital Medical University (YJ2021-05).

## Conflict of interest

The authors declare that the research was conducted in the absence of any commercial or financial relationships that could be construed as a potential conflict of interest.

## Publisher’s note

All claims expressed in this article are solely those of the authors and do not necessarily represent those of their affiliated organizations, or those of the publisher, the editors and the reviewers. Any product that may be evaluated in this article, or claim that may be made by its manufacturer, is not guaranteed or endorsed by the publisher.

## Abbreviations

SB: Suicidal behavior
MD: mood disorder
LASSO: least absolute shrinkage and selection operator
BMI: Body mass index
